# Anodal Stimulation of the Left DLPFC Increases IGT Scores and Decreases Delay Discounting Rate in Healthy Males

**DOI:** 10.3389/fpsyg.2016.01421

**Published:** 2016-09-20

**Authors:** Qinghua He, Mei Chen, Chuansheng Chen, Gui Xue, Tingyong Feng, Antoine Bechara

**Affiliations:** ^1^Decision Neuroscience Lab, Faculty of Psychology, Southwest UniversityChongqing, China; ^2^Key Laboratory of Cognition and Personality, Ministry of Education, Southwest UniversityChongqing, China; ^3^Key Laboratory of Mental Health, Institute of Psychology, Chinese Academy of SciencesBeijing, China; ^4^Southwest University Branch, Collaborative Innovation Center of Assessment Toward Basic Education Quality at Beijing Normal UniversityChongqing, China; ^5^Department of Psychology and Social Behavior, University of California at Irvine, IrvineCA, USA; ^6^National Key Laboratory of Cognitive Neuroscience and Learning, IDG/McGovern Institute for Brain Research, Beijing Normal UniversityBeijing, China; ^7^Brain and Creativity Institute and Department of Psychology, University of Southern California, Los AngelesCA, USA

**Keywords:** decision making, HD-tDCS, DLPFC, IGT, inter-temporal choice task

## Abstract

Previous correlational imaging studies have implicated the dorsolateral prefrontal cortex (DLPFC) in decision making. Using High-Definition Transcranial Direct Current Stimulation (HD-tDCS), the present study directly investigated the causal role of the DLPFC in performing the Iowa Gambling Task (IGT) and the Inter-Temporal Choice (ITC) task. Three experiments were conducted: Experiment 1 (*N* = 41) to study the left DLPFC, Experiment 2 (*N* = 49) to study the right DLPFC, and Experiment 3 (*N* = 20, a subset of those in Experiment 1) to switch the experimental and control conditions. All participants were healthy male college students. For Experiments 1 and 2, participants were randomly assigned to either the HD-tDCS or the sham stimulation condition. For Experiment 3, participants were assigned to the condition they were not in during Experiment 1. Results showed that HD-tDCS over the left DLPFC increased IGT score, decreased the recency parameter in IGT, and lowered delay discounting rate (*k*) in the ITC task. We discussed the potential roles of impulse control and time perception in mediating the effect of tDCS stimulation of left DLPFC on decision making. Our results have clinical implications for the treatment of disorders involving poor decision-making, such as addictions.

## Introduction

Decision making requires a trade-off between gains and losses (Levin et al., 2012). Behavioral economists have argued that good decision making is to maximize our expected utility over the long term (Summerfield and Tsetsos, 2015). Researchers have developed laboratory tasks to assess decision-making abilities. The Iowa Gambling Task (IGT) is probably the most commonly used experimental paradigm in clinical settings to assess the ability to sacrifice immediate interests in favor of long-term benefits (Bechara et al., 1994). On this task, participants choose one deck from four and over time normal participants gradually learn to choose from the two advantageous decks and avoid the two disadvantageous decks. The other commonly used task, especially among behavioral economists, is the Inter-Temporal Choice task (ITC), on which participants choose between a small immediate reward and a larger later one. In this task, future outcomes are devalued as a function of delay, which is referred to as the delay discounting (Peters and Buchel, 2011) and is well described by a hyperbolic discount function (Kirby and Herrnstein, 1995; Myerson and Green, 1995; Roelofsma, 1996; Rachlin et al., 2000).

Mounting evidence has suggested that the dorsolateral prefrontal cortex (DLPFC) plays an important role in decision making. For example, using either block design (Li X. et al., 2009) or event-related design (He et al., 2014), functional magnetic resonance imaging (fMRI) studies have revealed bilateral DLPFC activation during the decision-making stage of the IGT. Activation of the left DLPFC has also been linked to delay discounting in the intertemporal choice task (Weber and Huettel, 2008; Xu et al., 2009; Liu et al., 2012). Finally, DLPFC is activated by everyday decision making such as smoking (Kober et al., 2010) and food choice (Hare et al., 2009) as well as the ultimatum game (Sanfey et al., 2003).

However, the above studies only showed correlational results. Causal relationship between DLPFC and decision making still needs to be established. In a pioneering study, Figner et al. (2010) applied low-frequency repetitive transcranial magnetic stimulation (rTMS) to the left DLPFC and found that disrupting that region led to a preference for more immediate but smaller rewards over delayed but larger rewards. To extend this line of research, the present study used the High-definition Transcranial Direct Current Stimulation (HD-tDCS) to investigate the role of left and right DLPFC in decision making as assessed by the IGT and intertemporal choice task. The tDCS is a proven method of delivering a non-invasive brain stimulation (Gandiga et al., 2006). In three experiments, participants of this study completed the IGT, the ITC task, and the Barratt Impulsivity Scale (BIS) after either real or sham stimulation over the left or right DLPFC. We hypothesized that tDCS of the left DLPFC and/or the right DLPFC would lead to higher scores on the IGT and lower delay-discounting rates as compared to the sham stimulation.

In this study, we only recruited male subjects because of well-documented gender differences in the performance on decision making tasks, especially the IGT. Males are more likely than females to choose cards from the advantageous decks (Reavis and Overman, 2001; Overman et al., 2006; Weller et al., 2010; van den Bos et al., 2013). Neuroimaging studies also showed that brain regions activated by the IGT differed by gender, with females showing more left-lateralized brain activity than males (Bolla et al., 2004; van den Bos et al., 2009). There is also evidence of a gender-by-5-HTTLPR genotype interaction effect on IGT performance (He et al., 2010; Stoltenberg and Vandever, 2010).

## Experiment 1

### Participants

Forty-one healthy male college students volunteered to participate in this experiment. All participants had normal or corrected-to-normal vision. Their mean age was 20.7 years (*SD* = 1.59, range = 18–25). Based on the Structured Clinical Interview for DSM-IV (SCID), no participant met the criteria for psychotic, anxiety, bipolar, or substance abuse disorders. All participants gave informed consent to the experiment procedures, which were approved by the Southwest University Institutional Review Board. Participants were randomly assigned to either HD-tDCS over the left DLPFC region (*N* = 22) or sham stimulation to the same region (*N* = 19). All participants were naïve to tDCS research, and were asked not to consume any coffee or alcoholic beverages 2 h before the experiment. Each participant was paid 50 yuan for participation, regardless of his/her performance on the decision-making tasks.

### Design and Procedure

This study employed a single-blind between-subject design with random assignment of the participants. Recently developed HD-tDCS was used to deliver non-invasive anodal stimulation or sham stimulation to the left DLPFC. HD-tDCS has higher accuracy of current delivery by using 4 × 1 ring configuration instead of larger pad-electrodes of conventional tDCS. The safety and tolerability of HD-tDCS have been extensively tested (Villamar et al., 2013). This study followed all procedures of using HD-tDCS as demonstrated in Villamar et al. (2013). In brief, HD-tDCS was delivered by connecting a 4 × 1 multichannel stimulation adapter and a conventional tDCS device. A battery-driven current stimulator (Soterix Medical Inc., New York, NY, USA) was used to deliver a constant current. For participants receiving real HD-tDCS over the left DLPFC, the anodal electrode was placed over F3 based on the International 10–20 EEG System (Boggio et al., 2010; Gill et al., 2015) and the four return (cathodal) electrodes were placed over F5, AF3, FC3, and F1, around the active electrode. F3 was used as the anodal electrode location because most previous studies have used this location to stimulate the left DLPFC (Ambrus et al., 2011; Gill et al., 2015; Nieratschker et al., 2015). After the modular EEG recording cap was placed on the subject’s head, five plastic casings were fitted to their exact locations. Approximately 1.5 ml electrically conductive gel (Sigma Gel) was introduced through the opening of each plastic casing, beginning at the scalp surface. One Ag/AgCl sintered ring electrode was then placed in each HD plastic casing. The resistance impedance value of each electrode was verified to be less than or equal to 1.5–2.0 “quality units” (Villamar et al., 2013). The HD-tDCS was delivered at 1.5 mA for 20 min while participants read the instructions about the behavioral tasks. There was a ramp up and ramp down period of 30 s at the start and end of HD-tDCS. After the stimulation, participants completed the following behavioral tasks and questionnaire in the same order: (1) the IGT; (2) the ITC task; and (3) the BIS. At the end of the experiment, participants were verified on their tolerance of the experiment settings as well as their knowledge of the purpose of the study. Sham stimulation was conducted with the same montage, with 30 s of HD-tDCS applied at onset, after which the current stimulator was de-ramped.

### Behavioral Measures

#### The Iowa Gambling Task (IGT)

Participants were asked to complete the IGT, a computerized task used to test decision making under ambiguity and risk. The detail of this task has been extensively described in previous studies (Bechara et al., 1994, 1999). Briefly, four decks of cards labeled A, B, C, and D were displayed on the computer screen. Participants were asked to select one card at a time from one of the four decks. After each selection, a message was displayed on the screen indicating the payoff of that trial. Each card selection could bring an immediate reward (the immediate reward was higher in decks A and B than in decks C and D). As the game progressed, there were also unpredictable losses associated with each deck. The total losses were on average higher in decks A and B relative to decks C and D, thus creating a conflict in each choice, i.e., decks A and B are disadvantageous in the long run (even though they bring higher immediate reward), whereas decks C and D are advantageous in the long term (i.e., the long-term losses are smaller than the short-term gains, thus yielding a net profit). Net decision-making scores were obtained by subtracting the total number of selections from the disadvantageous decks (A and B) from the total number selections from the advantageous decks (C and D). Thus, positive numbers reflect good decisions, while negative numbers reflect bad decisions.

Following our previous studies (He et al., 2010, 2012; Koritzky et al., 2013), the IGT score was calculated for every 20 trials by subtracting the total number of disadvantageous deck selection from the total number of advantageous deck selection [i.e., (C + D - A - B)]. Then, following He et al. (2012) and Koritzky et al. (2013), the revised Expectancy Valence Model (rEV; Busemeyer and Stout, 2002; Yechiam et al., 2005) was used to model each participant’s choice on the IGT. Three parameters were generated for each participant (for details of the model, see Busemeyer and Stout, 2002; Yechiam et al., 2005): (1) Reward sensitivity (*W*), ranging from 0 to 1, with higher values denoting increased attention to gains over losses; (2) Recency (ϕ), ranging from 0 to 1, with higher values indicating rapid discounting of past outcomes; (3) Choice consistency (*c*), ranging from -5–5, with higher values representing converging choices toward the decks with the maximum reward expectancy. Detailed information on how to calculate these three parameters can be found in the Supplementary Materials. Following Yechiam et al. (2008), the rEV model was compared with a baseline model that simply used the average choices of preceding trials to predict the next choice (Busemeyer and Stout, 2002).

Bayesian Information Criterion was used for model comparison, with positive values of the difference in BIC (or dBIC) indicating that the rEV model performed better than the baseline model.

#### The Inter-temporal Choice Task (ITC)

The ITC asked participants to choose between small-but-sooner and larger-but-later rewards. For example, participants were asked to choose between receiving $50 now (option 1) or $54 in 2 weeks (option 2). A hyperbolic discount function provides a good estimate of delay discounting (Mazur, 1987, 1988): V = 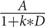, Where V is subjective value, *A* is the actual amount of money (i.e., $50 in option 1 and $54 in option 2), *D* is delay in days (i.e., 0 in option 1 and 14 in option 2), and *k* is the discounting rate, a subject-specific constant that quantifies delay discounting. *k* is a fit parameter that controls the steepness of the discount function, with *k* = 0 indicating no delay discounting and higher values of *k* indicating steeper discounting (Mazur, 1987; Steinbeis et al., 2016).

In our task, participants were first asked to complete the inter-temporal choice questionnaire (Kirby et al., 1999) on the computer, which would generate an initial discount factor *k*. The questionnaire had 27 items asking participants to choose between an immediate reward and a future reward. The amount of reward ranged from 11 Yuan to 85 Yuan, and the delay ranged from 7 days to 186 days. Then, participants were asked to complete an adaptive inter-temporal choice task on the computer with the initial *k*-value estimated from the questionnaire as the starting point. The task was adapted from Kirby et al. (1999) and Luo et al. (2009, 2012). The adaptive procedure allowed for a precise estimation of *k*. The delay was always 120 days, and the magnitude of the future reward was held constant. The initial amount of the immediate reward was generated by computing what would be an amount of equal value to the future reward based on the *k* parameter derived from the questionnaire. For each trial, if the participant chose the immediate reward, the *k* parameter was adjusted upward by a quarter step on a *log_10_* scale, and consequently, the value of the immediate reward on the next trial was lower. Conversely, if the participant chose the future reward, then the *k* parameter was adjusted downward by a quarter step on a *log_10_* scale, resulting in a larger immediate reward on the next trial. This adaptive procedure continued until *k* did not deviate by more than two steps in a window of eight trials. All participants reached this criterion. Filler trials were inserted in the sequence to prevent participants from noticing the adjustment. The final indifference pairs were then generated using the geometric mean of the *k*-values for the eight trials during which stabilization was achieved. Because the stable *k* parameter was not normally distributed, it was natural-log transformed for group analysis.

#### The Barratt Impulsivity Scale (BIS)

Participants were also asked to complete the BIS (Jollant et al., 2005; Zermatten et al., 2005; Franken et al., 2008; Wittmann and Paulus, 2008), which is a 30-item questionnaire assessing three components of impulsivity: attentional impulsivity, motor impulsivity, and non-planning impulsivity. Participants rated each item on a 4-point scale (Rarely/Never = 1; Occasionally = 2; Often = 3; Almost Always/Always = 4). The scale had good reliability in this study (*Cronbach’s Alpha* = 0.783 for the whole scale and >0.717 for each subscale).

### Results

The mean ages of the two groups of subjects were comparable [HD-tDCS group = 20.5 ± 1.63 years, Sham group = 20.8 ± 1.57 years, *t*(39) = 0.68, *p* = 0.50]. Their scores of attention impulsivity [HD-tDCS group = 3.7 ± 0.29, Sham group = 3.8 ± 0.41, *t*(39) = 0.36, *p* = 0.72], motor impulsivity [HD-tDCS group = 2.3 ± 0.46, Sham group = 2.2 ± 0.43, *t*(39) = 0.07, *p* = 0.94], and non-planning impulsivity [HD-tDCS group = 3.7 ± 0.46, Sham group = 3.7 ± 0.59, *t*(39) = 0.06, *p* = 0.95] were also comparable.

**FIGURE 1:**
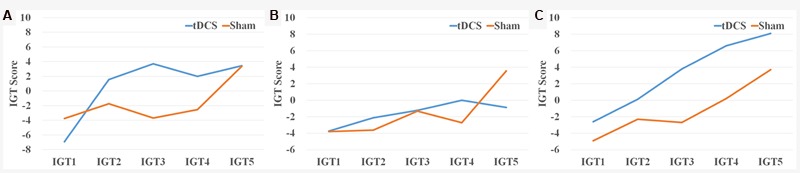
**IGT scores by block and condition for the **(A)** experiment 1, **(B)** experiment 2, and **(C)** experiment 3**.

Iowa Gambling Task scores were first analyzed by five blocks of 20 trials following the procedure used in previous studies (Bechara et al., 1994). A 5 (blocks of IGT, a within-subject factor) × 2 (group: HD-tDCS vs. Sham, a between-subject factor) ANOVA revealed a significant main effect of IGT block [*F*(4,156) = 7.65, *p* = 0.0001, $\eta_{p}^{2}$ = 0.16], suggesting that participants were more likely to select from the advantageous decks as the task progressed. Although there was no main effect of group [*F*(1,39) = 1.22, *p* = 0.28], there was a significant interaction between IGT block and group [*F*(4,156) = 3.27, *p* = 0.01, $\eta_{p}^{2}$ = 0.08]. As illustrated in **Figure 1**, the HD-tDCS group showed faster learning than the sham group, and the biggest difference was for the IGT scores in the trials 41–60 [*t*(39) = 2.68, *p* = 0.01, Cohen’s *d* = 0.86] (**Figure 1**).

**FIGURE 2:**
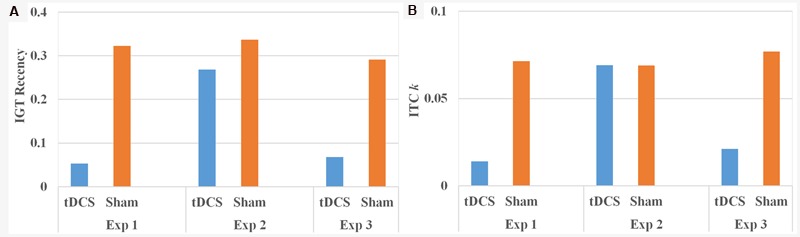
**IGT recency parameter **(A)** and ITC *k*-value **(B)** by condition for the three experiments**.

The rEV model provided a better fit than the baseline model to the IGT performance (HD-tDCS group dBIC = 18.39 ± 4.67, Sham group dBIC = 18.58 ± 4.83), and there was no difference in dBIC between the two groups [*t*(39) = 0.13, *p* = 0.55]. Three parameters estimated from the rEV model were compared between the two groups using independent-samples *t*-test. Results suggested a significant group difference in the recency parameter [*t*(39) = 3.02, *p* = 0.004, Cohen’s *d* = 0.97] (**Figure 2**) but no group differences in the other two parameters (both *t*s < 0.37, *p*s > 0.71). The HD-tDCS group (0.05 ± 0.08) had a smaller recency parameter than the sham group (0.32 ± 0.40), suggesting that the HD-tDCS group relied more on the past information and learned faster than did the sham group. Because the recency parameter had a skewed distribution, we re-analyzed the data after a natural log transformation and found similar results, [*t*(39) = 3.44, *p* = 0.001, Cohen’s *d* = 1.10].

The *k*-value from the ITC task also showed a skewed distribution. After a natural log transformation of the final *k*-value, independent-samples *t*-test showed a significant group difference [*t*(39) = 2.34, *p* = 0.02, Cohen’s *d* = 0.75] (**Figure 2**), with the HD-tDCS group (0.01 ± 0.01) having a smaller *k*-value than the sham group (0.07 ± 0.05).

## Experiment 2

### Experimental Settings

Participants were 49 healthy male college students (mean age = 20.5 years, *SD* = 1.63, range = 18–25) who did not participate in Experiment 1. These participants met all the inclusion criteria described in Experiment 1. The design and procedure were exactly the same as Experiment 1 except that the location of the anodal/sham stimulation was the right DLPFC region (23 participants received anodal stimulation and 26 received sham stimulation). The BIS scale also had good reliability in this experiment (*Cronbach’s Alpha* = 0.832 for the whole scale and >0.753 for each subscale).

### Results

The mean ages of the two groups were comparable [HD-tDCS group = 20.3 ± 1.68 years, Sham group = 20.7 ± 1.55 years, *t*(47) = 0.52, *p* = 0.61]. Their scores of attentional impulsivity [HD-tDCS group = 3.8 ± 0.49, Sham group = 3.9 ± 0.29, *t*(47) = 0.62, *p* = 0.54], motor impulsivity [HD-tDCS group = 2.2 ± 0.61, Sham group = 2.3 ± 0.43, *t*(47) = 1.01, *p* = 0.32], and non-planning impulsivity [HD-tDCS group = 3.8 ± 0.55, Sham group = 3.6 ± 0.37, *t*(47) = 1.24, *p* = 0.22] were also comparable.

Iowa Gambling Task scores were first analyzed by five blocks of 20 trials as described in Experiment 1. A 5 (blocks of IGT, a within-subject factor) × 2 (group: HD-tDCS vs. Sham, a between-subject factor) ANOVA revealed a significant main effect of IGT block [*F*(4,188) = 4.51, *p* = 0.002, $\eta_{p}^{2}$ = 0.09], but no main effect of group [*F*(1,47) = 0.01, *p* = 0.99] or interaction between IGT block and group [*F*(4,188) = 2.16, *p* = 0.15] (**Figure 1**). The rEV model provided a better fit than the baseline model to the IGT performance (HD-tDCS group dBIC = 16.68 ± 4.77, Sham group dBIC = 16.53 ± 4.29), and there was no difference in dBIC between the two groups [*t*(47) = 0.12, *p* = 0.55]. There was also no effect of HD-tDCS on either IGT parameters estimated from the rEV model (all *t*s < 1.47, *p*s > 0.15) or *k*-value from the ITC task [*t*(47) = 0.01, *p* = 0.99] (**Figure 2**).

Finally, the sham conditions across two experiments were compared. A 5 (blocks of IGT, a within-subject factor) × 2 (experiments, a between-subject factor) ANOVA revealed no main effect of experiment on IGT scores [*F*(1,43) = 0.002, *p* = 0.96] and no interaction between IGT block and experiment [*F*(1,172) = 0.56, *p* = 0.70]. There was also no difference between the two experiments’ sham conditions in the recency parameter [*t*(43) = 1.57, *p* = 0.13], *k* [*t*(43) = 0.97, *p* = 0.92], and BIS scores (all *t*s < 1.21, all *p*s > 0.23).

## Experiment 3

### Experimental Settings

Twenty participants (mean age = 19.7 ± 0.92 years, ranging from 18 to 21) who participated in Experiment 1 were recruited for this experiment. If they had their HD-tDCS over the left DLPFC in Experiment 1, they were assigned to the sham condition for Experiment 3 (*N* = 11); if they were in the sham group in Experiment 1, they were assigned to the HD-tDCS condition in Experiment 3 (*N* = 9). The BIS scale had a good reliability for the second administration to this subgroup (*Cronbach’s alpha* = 0.865 for the whole scale and >0.763 for each subscale). The correlations between the first (Experiment 1) and the second (Experiment 3) administrations (test–retest reliability) were high for the whole scale (*r* = 0.63, *p* = 0.002) and the three subscales (attentional impulsivity: *r* = 0.65, *p* = 0.001; motor impulsivity: *r* = 0.64, *p* = 0.001; and non-planning impulsivity: *r* = 0.67, *p* = 0.0007).

### Results

The two groups did not differ significantly in their second assessment of attentional impulsivity [HD-tDCS group (labeled based on their condition in Experiment 3) = 3.7 ± 0.33, Sham group = 3.8 ± 0.29, *t*(19) = 1.47, *p* = 0.16], motor impulsivity [HD-tDCS group = 2.2 ± 0.41, Sham group = 2.3 ± 0.50, *t*(19) = 0.73, *p* = 0.48], and non-planning impulsivity [HD-tDCS group = 3.7 ± 0.33, Sham group = 3.6 ± 0.47, *t*(19) = 1.48, *p* = 0.17].

Because all participants in Experiment 3 came from Experiment 1, we analyzed the data as a within-subject design. A 5 (blocks of IGT, a within-subject factor) × 2 (condition: HD-tDCS vs. Sham, a within-subject factor) × 2 (order: HD-tDCS condition first vs. Sham condition first, a between-subject factor) ANOVA revealed a significant main effect of IGT block [*F*(4,72) = 14.33, *p* = 0.0001, $\eta_{p}^{2}$ = 0.44] and a significant main effect of condition [*F*(1,18) = 6.89, *p* = 0.017, $\eta_{p}^{2}$ = 0.28] (**Figure 1**), but no main effect of order [*F*(1,18) = 0.72, *p* = 0.41], no interaction between IGT block and condition [*F*(4,72) = 0.97, *p* = 0.43], no interaction between IGT block and order [*F*(4,72) = 0.77, *p* = 0.55], no interaction between condition and order [*F*(1,18) = 0.41, *p* = 0.53], and no three-way interaction [*F*(4,72) = 1.01, *p* = 0.41].

Because the order of the conditions was not a significant factor in the above analysis, the rEV model was fitted to the two conditions with data pooled from both Experiments 1 and 3. The rEV model provided a better fit than the baseline model to the IGT performance in both conditions (HD-tDCS condition dBIC = 18.83 ± 4.32, Sham condition dBIC = 18.68 ± 4.52), and there was no difference in dBIC between the two conditions [*t*(19) = 0.16, *p* = 0.56]. Three parameters estimated from rEV model were compared between the two conditions using paired-samples *t-*test. Results showed a significant condition difference in the recency parameter [*t*(19) = 2.57, *p* = 0.02, Cohen’s *d* = 1.28] (**Figure 2**), but no differences were found in the other two parameters (both *t*s < 0.23, *p*s > 0.80). The HD-tDCS condition (0.11 ± 0.03) had a smaller recency parameter than the sham condition (0.39 ± 0.09), suggesting that after HD-tDCS, subjects relied more on the past information and learned faster as compared to after the sham stimulation. Because the recency parameter had a skewed distribution, we re-analyzed the data after a natural log transformation and found similar results, [*t*(19) = 2.83, *p* = 0.01, Cohen’s *d* = 1.42].

The *k*-value from the ITC task also showed a skewed distribution. After a natural log transformation of the final *k*-value, paired-samples *t*-test showed a significant condition difference [*t*(19) = 2.35, *p* = 0.03, Cohen’s *d* = 0.89], with the HD-tDCS condition (0.02 ± 0.02) having a smaller *k*-value than the sham condition (0.07 ± 0.06) (**Figure 2**).

## Discussion

The objective of this study was to investigate the role of DLPFC in decision making as assessed by the IGT and the ITC task. Using high-definition anodal tDCS stimulation over the left or right DLPFC, we found that stimulation over the left, but not that over the right DLPFC led to higher IGT score (especially for the middle 20 trials), lower recency parameter based on the rEV model, and lower delay-discounting rate (*k*). The stimulation did not influence the personality trait of impulsivity as measured by the BIS.

Our results are consistent with three lines of evidence suggesting that the left DLPFC is essential for decision making. First, lesion studies have suggested that damage to the left DLPFC would compromise decision making (Bechara et al., 1998; Manes et al., 2002; Fellows and Farah, 2005). For example, Manes et al. (2002) found that patients with left DLPFC damage showed pronounced impairment on the IGT, as well as on working memory, planning, and attentional shifting tasks. Second, neuroimaging studies have indicated that the left DLPFC is involved in the decision making process (Rorie and Newsome, 2005; Heekeren et al., 2006). fMRI studies have revealed left DLPFC activation during the decision making stage of the IGT (Li X. et al., 2009; Lin et al., 2012; He et al., 2014). The activation of DLPFC during the execution stage of the IGT was associated with choice risk level in an functional near-infrared spectroscopy (fNIRS) study (Bembich et al., 2014). Moreover, later fMRI studies even showed that the activity of DLPFC was inversely correlated with future reward delay (McClure et al., 2004; Kable and Glimcher, 2007; Ballard and Knutson, 2009). Third, using brain stimulation techniques, preliminary studies have shown a causal role of left DLPFC in decision making (Figner et al., 2010; Philiastides et al., 2011). For example, Philiastides et al. (2011) showed that disruption of the left DLPFC with low-frequency rTMS influenced perceptual decision making. In the present study, non-invasive brain stimulation with HD-tDCS was applied to increase cortical excitability in DLPFC. For the first time, we found a causal role of left DLPFC in both risky decision making and inter-temporal choice.

Several cognitive mechanisms might have been involved in the positive effect of left DLPFC stimulation on decision making. One is impulse control, which may be temporarily elevated. Mounting evidence has suggested that in addition to its role in decision making as mentioned earlier, DLPFC also plays an important role in impulse control (Li C.S. et al., 2009; Boggio et al., 2010; Weygandt et al., 2013). For example, higher DLPFC activity has been linked to greater inhibition control in a study of successful dietary restraints (Weygandt et al., 2013). Loss of DLPFC function has been linked to a lower level of inhibition control in research on addictions (Crews and Boettiger, 2009). Our study measured the personality trait of impulse control, which did not seem to be affected by HD-tDCS over the left DLPFC, perhaps because it was a relatively stable personality trait. Future studies should directly measure the inhibition control ability and examine its potential mediating role in the effect of HD-tDCS on decision making. If such a mediating role is confirmed, HD-tDCS over the left DLPFC can have potential clinical applications because a deficit in inhibition control underlies most types of addictions (Noël et al., 2013).

Another possible mechanism involved in left DLPFC’s role in decision making is time perception. Previous research has shown that left DLPFC is activated by the processing of temporal information (Hinton et al., 1996; Rao et al., 2001; Basso et al., 2003; Nenadic et al., 2003). Perhaps HD-tDCS over this brain region helped the participants gain a more appropriate time perspective, and consequently reducing their temporal discount. Indeed, previous studies have already shown that activity in left DLPFC was positively associated with a preference for delayed rewards (McClure et al., 2007; Christakou et al., 2011; Civai et al., 2016) and lower delay discounting rates (Hayashi et al., 2013; Sheffer et al., 2013). Although previous discussions about time perspective in decision making focused on future discounting, it also seems appropriate to consider time perspective when examining decision making based on past events (e.g., IGT). To some extent, the recency parameter of IGT reflects participants’ relative attention to the more recent as compared to more distant past outcomes. Future studies should test the above conjecture by directly measuring temporal perception after stimulating the left DLPFC.

Three limitations of this study should be noted. First, the sample used for our study included only males. Our results may or may not generalize to females because there are well-documented gender differences in decision making. Second, we failed to replicate the finding from previous studies that right DLPFC also plays an important role in decision making (Essex et al., 2012). Perhaps the right DLPFC is normally involved in response inhibition (Aron et al., 2003, 2004, 2014), rather than impulse control in decision making (Miller and Cohen, 2001; Steinbeis et al., 2012). Third, it is not clear how long the effect of the HD-tDCS would last, which is an important issue in clinical applications. Some studies reported that the effect of tDCS was relatively short (Goldman et al., 2011), but others found that the effect could last for a longer time (Nitsche and Paulus, 2000, 2001).

## Conclusion

The present study investigated the role of DLPFC in decision making as assessed by the IGT and the ITC task. Using high-definition anodal tDCS stimulation over the DLPFC, we found that stimulation over the left but not that over the right DLPFC led to higher IGT scores, lower recency parameter based on the rEV model, and lower delay discounting rate. The stimulation did not influence the personality trait of impulsivity as measured by the BIS. Based on our results as well as previous research, we speculated that impulse control and/or time perspective might be the mediating processes between the stimulation of left DLPFC and decision making. If future research confirms the role of HD-tDCS over left DLPFC in impulse control, such stimulation may have potential clinical implications for the treatment of addictions.

## Author Contributions

Conceived and designed the experiments: QH, GX, and AB. Performed the experiments: QH and MC. Analyzed the data: QH and MC. Wrote the paper: QH, MC, CC, GX, TF, and AB. All authors read and approved the final manuscript.

## Conflict of Interest Statement

The authors declare that the research was conducted in the absence of any commercial or financial relationships that could be construed as a potential conflict of interest.

## References

[B1] Ambrus G. G. Zimmer M. Kincses Z. T. Harza I. Kovacs G. Paulus W. (2011). The enhancement of cortical excitability over the DLPFC before and during training impairs categorization in the prototype distortion task. *Neuropsychologia* 49 1974–1980. 10.1016/j.neuropsychologia.2011.03.026 21440562

[B2] Aron A. R. Fletcher P. C. Bullmore E. T. Sahakian B. J. Robbins T. W. (2003). Stop-signal inhibition disrupted by damage to right inferior frontal gyrus in humans. *Nat. Neurosci.* 6 115–116. 10.1038/nn1003 12536210

[B3] Aron A. R. Robbins T. W. Poldrack R. A. (2004). Inhibition and the right inferior frontal cortex. *Trends Cogn. Sci.* 8 170–177. 10.1016/j.tics.2004.02.010 15050513

[B4] Aron A. R. Robbins T. W. Poldrack R. A. (2014). Inhibition and the right inferior frontal cortex: one decade on. *Trends Cogn. Sci.* 18 177–185. 10.1016/j.tics.2013.12.003 24440116

[B5] Ballard K. Knutson B. (2009). Dissociable neural representations of future reward magnitude and delay during temporal discounting. *Neuroimage* 45 143–150. 10.1016/j.neuroimage.2008.11.004 19071223 PMC2685201

[B6] Basso G. Nichelli P. Wharton C. M. Peterson M. Grafman J. (2003). Distributed neural systems for temporal production: a functional MRI study. *Brain Res. Bull.* 59 405–411. 10.1016/S0361-9230(02)00941-3 12507693

[B7] Bechara A. Damasio A. R. Damasio H. Anderson S. W. (1994). Insensitivity to future consequences following damage to human prefrontal cortex. *Cognition* 50 7–15. 10.1016/0010-0277(94)90018-3 8039375

[B8] Bechara A. Damasio H. Damasio A. R. Lee G. P. (1999). Different contributions of the human amygdala and ventromedial prefrontal cortex to decision-making. *J. Neurosci.* 19 5473–5481. 10377356 10.1523/JNEUROSCI.19-13-05473.1999 PMC6782338

[B9] Bechara A. Damasio H. Tranel D. Anderson S. W. (1998). Dissociation of working memory from decision making within the human prefrontal cortex. *J. Neurosci.* 18 428–437. 9412519 10.1523/JNEUROSCI.18-01-00428.1998 PMC6793407

[B10] Bembich S. Clarici A. Vecchiet C. Baldassi G. Cont G. Demarini S. (2014). Differences in time course activation of dorsolateral prefrontal cortex associated with low or high risk choices in a gambling task. *Front. Hum. Neurosci.* 8:464 10.3389/fnhum.2014.00464 PMC4067729 25009486

[B11] Boggio P. S. Zaghi S. Villani A. B. Fecteau S. Pascual-Leone A. Fregni F. (2010). Modulation of risk-taking in marijuana users by transcranial direct current stimulation (tDCS) of the dorsolateral prefrontal cortex (DLPFC). *Drug Alcohol Depend.* 112 220–225. 10.1016/j.drugalcdep.2010.06.019 20729009

[B12] Bolla K. I. Eldreth D. A. Matochik J. A. Cadet J. L. (2004). Sex-related differences in a gambling task and its neurological correlates. *Cereb. Cortex* 14 1226–1232. 10.1093/cercor/bhh083 15142963

[B13] Busemeyer J. R. Stout J. C. (2002). A contribution of cognitive decision models to clinical assessment: decomposing performance on the Bechara gambling task. *Psychol. Assess.* 14 253–262. 10.1037/1040-3590.14.3.253 12214432

[B14] Christakou A. Brammer M. Rubia K. (2011). Maturation of limbic corticostriatal activation and connectivity associated with developmental changes in temporal discounting. *Neuroimage* 54 1344–1354. 10.1016/j.neuroimage.2010.08.067 20816974

[B15] Civai C. Hawes D. R. DeYoung C. G. Rustichini A. (2016). Intelligence and extraversion in the neural evaluation of delayed rewards. *J. Res. Pers.* 61 99–108. 10.1016/j.jrp.2016.02.006

[B16] Crews F. T. Boettiger C. A. (2009). Impulsivity, frontal lobes and risk for addiction. *Pharmacol. Biochem. Behav.* 93 237–247. 10.1016/j.pbb.2009.04.018 19410598 PMC2730661

[B17] Essex B. G. Clinton S. A. Wonderley L. R. Zald D. H. (2012). The impact of the posterior parietal and dorsolateral prefrontal cortices on the optimization of long-term versus immediate value. *J. Neurosci.* 32 15403–15413. 10.1523/JNEUROSCI.6106-11.2012 23115178 PMC6621579

[B18] Fellows L. K. Farah M. J. (2005). Different underlying impairments in decision-making following ventromedial and dorsolateral frontal lobe damage in humans. *Cereb. Cortex* 15 58–63. 10.1093/cercor/bhh108 15217900

[B19] Figner B. Knoch D. Johnson E. J. Krosch A. Lisanby S. H. Fehr E. (2010). Lateral prefrontal cortex and self-control in intertemporal choice. *Nat. Neurosci.* 13 538–539. 10.1038/nn.2516 20348919

[B20] Franken I. H. Van Strien J. W. Nijs I. Muris P. (2008). Impulsivity is associated with behavioral decision-making deficits. *Psychiatry Res.* 158 155–163. 10.1016/j.psychres.2007.06.002 18215765

[B21] Gandiga P. C. Hummel F. C. Cohen L. G. (2006). Transcranial DC stimulation (tDCS): a tool for double-blind sham-controlled clinical studies in brain stimulation. *Clin. Neurophysiol.* 117 845–850. 10.1016/j.clinph.2005.12.003 16427357

[B22] Gill J. Shah-Basak P. P. Hamilton R. (2015). It’s the thought that counts: examining the task-dependent effects of transcranial direct current stimulation on executive function. *Brain Stimul.* 8 253–259. 10.1016/j.brs.2014.10.018 25465291

[B23] Goldman R. L. Borckardt J. J. Frohman H. A. O’Neil P. M. Madan A. Campbell L. K. (2011). Prefrontal cortex transcranial direct current stimulation (tDCS) temporarily reduces food cravings and increases the self-reported ability to resist food in adults with frequent food craving. *Appetite* 56 741–746. 10.1016/j.appet.2011.02.013 21352881

[B24] Hare T. A. Camerer C. F. Rangel A. (2009). Self-control in decision-making involves modulation of the vmPFC valuation system. *Science* 324 646–648. 10.1126/science.1168450 19407204

[B25] Hayashi T. Ko J. H. Strafella A. P. Dagher A. (2013). Dorsolateral prefrontal and orbitofrontal cortex interactions during self-control of cigarette craving. *Proc. Natl. Acad. Sci. U.S.A.* 110 4422–4427. 10.1073/pnas.1212185110 23359677 PMC3600476

[B26] He Q. Xiao L. Xue G. Wong S. Ames S. L. Xie B. (2014). Altered dynamics between neural systems sub-serving decisions for unhealthy food. *Front. Neurosci.* 8:350 10.3389/fnins.2014.00350 PMC4220120 25414630

[B27] He Q. Xue G. Chen C. Lu Z. Dong Q. Lei X. (2010). Serotonin transporter gene-linked polymorphic region (5-HTTLPR) influences decision making under ambiguity and risk in a large Chinese sample. *Neuropharmacology* 59 518–526. 10.1016/j.neuropharm.2010.07.008 20659488 PMC2946467

[B28] He Q. Xue G. Chen C. Lu Z. L. Lei X. Liu Y. (2012). COMT Val158Met polymorphism interacts with stressful life events and parental warmth to influence decision making. *Sci. Rep.* 2:677 10.1038/srep00677 PMC3447184 22997551

[B29] Heekeren H. R. Marrett S. Ruff D. A. Bandettini P. Ungerleider L. G. (2006). Involvement of human left dorsolateral prefrontal cortex in perceptual decision making is independent of response modality. *Proc. Natl. Acad. Sci. U.S.A.* 103 10023–10028. 10.1073/pnas.0603949103 16785427 PMC1479865

[B30] Hinton S. Meck W. MacFall J. (1996). Peak-interval timing in humans activates frontal-striatal loops. *Neuroimage* 3:S224 10.1016/S1053-8119(96)80226-6

[B31] Jollant F. Bellivier F. Leboyer M. Astruc B. Torres S. Verdier R. (2005). Impaired decision making in suicide attempters. *Am. J. Psychiatry* 162 304–310. 10.1176/appi.ajp.162.2.304 15677595

[B32] Kable J. W. Glimcher P. W. (2007). The neural correlates of subjective value during intertemporal choice. *Nat. Neurosci.* 10 1625–1633. 10.1038/nn2007 17982449 PMC2845395

[B33] Kirby K. N. Herrnstein R. J. (1995). Preference reversals due to myopic discounting of delayed reward. *Psychol. Sci.* 6 83–89. 10.1111/j.1467-9280.1995.tb00311.x

[B34] Kirby K. N. Petry N. M. Bickel W. K. (1999). Heroin addicts have higher discount rates for delayed rewards than non-drug-using controls. *J. Exp. Psychol. Gen.* 128 78–87. 10.1037/0096-3445.128.1.78 10100392

[B35] Kober H. Mende-Siedlecki P. Kross E. F. Weber J. Mischel W. Hart C. L. (2010). Prefrontal-striatal pathway underlies cognitive regulation of craving. *Proc. Natl. Acad. Sci. U.S.A.* 107 14811–14816. 10.1073/pnas.1007779107 20679212 PMC2930456

[B36] Koritzky G. He Q. Xue G. Wong S. Xiao L. Bechara A. (2013). Processing of time within the prefrontal cortex: recent time engages posterior areas whereas distant time engages anterior areas. *Neuroimage* 72 280–286. 10.1016/j.neuroimage.2013.01.056 23380168

[B37] Levin I. P. Xue G. Weller J. A. Reimann M. Lauriola M. Bechara A. (2012). A neuropsychological approach to understanding risk-taking for potential gains and losses. *Front. Neurosci.* 6:15 10.3389/fnins.2012.00015 PMC3273874 22347161

[B38] Li C. S. Luo X. Yan P. Bergquist K. Sinha R. (2009). Altered impulse control in alcohol dependence: neural measures of stop signal performance. *Alcohol. Clin. Exp. Res.* 33 740–750. 10.1111/j.1530-0277.2008.00891.x 19170662 PMC2697053

[B39] Li X. Lu Z.-L. D’Argembeau A. Ng M. Bechara A. (2009). The Iowa Gambling Task in fMRI images. *Hum. Brain Mapp.* 31 410–423. 10.1002/hbm.20875 19777556 PMC2826566

[B40] Lin B. Qian R. Fu X. Ji X. Wei X. Niu C. (2012). [Impulsive decision-making behaviors in heroin addicts: a study of functional magnetic resonance imaging]. *Zhonghua yi xue za zhi* 92 1033–1036. 22781643

[B41] Liu L. Feng T. Wang J. Li H. (2012). The neural dissociation of subjective valuation from choice processes in intertemporal choice. *Behav. Brain Res.* 231 40–47. 10.1016/j.bbr.2012.02.045 22406016

[B42] Luo S. Ainslie G. Giragosian L. Monterosso J. R. (2009). Behavioral and neural evidence of incentive bias for immediate rewards relative to preference-matched delayed rewards. *J. Neurosci.* 29 14820–14827. 10.1523/JNEUROSCI.4261-09.2009 19940177 PMC2821568

[B43] Luo S. Ainslie G. Pollini D. Giragosian L. Monterosso J. R. (2012). Moderators of the association between brain activation and farsighted choice. *Neuroimage* 59 1469–1477. 10.1016/j.neuroimage.2011.08.004 21856429 PMC12842337

[B44] Manes F. Sahakian B. Clark L. Rogers R. Antoun N. Aitken M. (2002). Decision-making processes following damage to the prefrontal cortex. *Brain* 125 624–639. 10.1093/brain/awf049 11872618

[B45] Mazur J. E. (1987). “An adjusting procedure for studying delayed reinforcement,” in *Quantitative Analyses of Behavior: The Effect of Delay and of Intervening Events on Reinforcement Value* Vol. 5 eds Commons M. L. Mazur J. E. Nevin J. A. Rachlin H. (Hillsdale, NJ: Earlbaum) 55–73.

[B46] Mazur J. E. (1988). Estimation of indifference points with an adjusting-delay procedure. *J. Exp. Anal. Behav.* 49 37–47. 10.1901/jeab.1988.49-37 3346621 PMC1338825

[B47] McClure S. M. Ericson K. M. Laibson D. I. Loewenstein G. Cohen J. D. (2007). Time discounting for primary rewards. *J. Neurosci.* 27 5796–5804. 10.1523/JNEUROSCI.4246-06.2007 17522323 PMC6672764

[B48] McClure S. M. Laibson D. I. Loewenstein G. Cohen J. D. (2004). Separate neural systems value immediate and delayed monetary rewards. *Science* 306 503–507. 10.1126/science.1100907 15486304

[B49] Miller E. K. Cohen J. D. (2001). An integrative theory of prefrontal cortex function. *Annu. Rev. Neurosci.* 24 167–202. 10.1146/annurev.neuro.24.1.167 11283309

[B50] Myerson J. Green L. (1995). Discounting of delayed rewards: models of individual choice. *J. Exp. Anal. Behav.* 64 263–276. 10.1901/jeab.1995.64-263 16812772 PMC1350137

[B51] Nenadic I. Gaser C. Volz H.-P. Rammsayer T. Häger F. Sauer H. (2003). Processing of temporal information and the basal ganglia: new evidence from fMRI. *Exp. Brain Res.* 148 238–246. 12520413 10.1007/s00221-002-1188-4

[B52] Nieratschker V. Kiefer C. Giel K. Kruger R. Plewnia C. (2015). The COMT Val/Met polymorphism modulates effects of tDCS on response inhibition. *Brain Stimul.* 8 283–288. 10.1016/j.brs.2014.11.009 25496958

[B53] Nitsche M. Paulus W. (2000). Excitability changes induced in the human motor cortex by weak transcranial direct current stimulation. *J. Physiol.* 527 633–639. 10.1111/j.1469-7793.2000.t01-1-00633.x 10990547 PMC2270099

[B54] Nitsche M. A. Paulus W. (2001). Sustained excitability elevations induced by transcranial DC motor cortex stimulation in humans. *Neurology* 57 1899–1901. 10.1212/WNL.57.10.1899 11723286

[B55] Noël X. Brevers D. Bechara A. (2013). A neurocognitive approach to understanding the neurobiology of addiction. *Curr. Opin. Neurobiol.* 23 632–638. 10.1016/j.conb.2013.01.018 23395462 PMC3670974

[B56] Overman W. Graham L. Redmond A. Eubank R. Boettcher L. Samplawski O. (2006). Contemplation of moral dilemmas eliminates sex differences on the Iowa gambling task. *Behav. Neurosci.* 120 817–825. 10.1037/0735-7044.120.4.817 16893287

[B57] Peters J. Buchel C. (2011). The neural mechanisms of inter-temporal decision-making: understanding variability. *Trends Cogn. Sci.* 15 227–239. 10.1016/j.tics.2011.03.002 21497544

[B58] Philiastides M. G. Auksztulewicz R. Heekeren H. R. Blankenburg F. (2011). Causal role of dorsolateral prefrontal cortex in human perceptual decision making. *Curr. Biol.* 21 980–983. 10.1016/j.cub.2011.04.034 21620706

[B59] Rachlin H. Brown J. Cross D. (2000). Discounting in judgments of delay and probability. *J. Behav. Decis. Mak.* 13 145–159. 10.1002/(SICI)1099-0771(200004/06)13:2<145::AID-BDM320>3.0.CO;2-4

[B60] Rao S. M. Mayer A. R. Harrington D. L. (2001). The evolution of brain activation during temporal processing. *Nat. Neurosci.* 4 317–323. 10.1038/85191 11224550

[B61] Reavis R. Overman W. H. (2001). Adult sex differences on a decision-making task previously shown to depend on the orbital prefrontal cortex. *Behav. Neurosci.* 115 196–206. 10.1037/0735-7044.115.1.196 11256443

[B62] Roelofsma P. H. (1996). Modelling intertemporal choices: an anomaly approach. *Acta Psychol.* 93 5–22. 10.1016/0001-6918(96)00023-6

[B63] Rorie A. E. Newsome W. T. (2005). A general mechanism for decision-making in the human brain? *Trends Cogn. Sci.* 9 41–43. 10.1016/j.tics.2004.12.007 15668095

[B64] Sanfey A. G. Rilling J. K. Aronson J. A. Nystrom L. E. Cohen J. D. (2003). The neural basis of economic decision-making in the Ultimatum Game. *Science* 300 1755–1758. 10.1126/science.1082976 12805551

[B65] Sheffer C. E. Mennemeier M. Landes R. D. Bickel W. K. Brackman S. Dornhoffer J. (2013). Neuromodulation of delay discounting, the reflection effect, and cigarette consumption. *J. Subst. Abuse Treat.* 45 206–214. 10.1016/j.jsat.2013.01.012 23518286 PMC3690153

[B66] Steinbeis N. Bernhardt B. C. Singer T. (2012). Impulse control and underlying functions of the left DLPFC mediate age-related and age-independent individual differences in strategic social behavior. *Neuron* 73 1040–1051. 10.1016/j.neuron.2011.12.027 22405212

[B67] Steinbeis N. Haushofer J. Fehr E. Singer T. (2016). Development of Behavioral control and associated vmPFC–DLPFC connectivity explains children’s increased resistance to temptation in intertemporal choice. *Cereb. Cortex* 26 32–42. 10.1093/cercor/bhu167 25100855

[B68] Stoltenberg S. F. Vandever J. M. (2010). Gender moderates the association between 5-HTTLPR and decision-making under ambiguity but not under risk. *Neuropharmacology* 58 423–428. 10.1016/j.neuropharm.2009.09.010 19781560 PMC3107605

[B69] Summerfield C. Tsetsos K. (2015). Do humans make good decisions? *Trends Cogn. Sci.* 19 27–34. 10.1016/j.tics.2014.11.005 25488076 PMC4286584

[B70] van den Bos R. Harteveld M. Stoop H. (2009). Stress and decision-making in humans: performance is related to cortisol reactivity, albeit differently in men and women. *Psychoneuroendocrinology* 34 1449–1458. 10.1016/j.psyneuen.2009.04.016 19497677

[B71] van den Bos R. Homberg J. de Visser L. (2013). A critical review of sex differences in decision-making tasks: focus on the Iowa Gambling Task. *Behav. Brain Res.* 238 95–108. 10.1016/j.bbr.2012.10.002 23078950

[B72] Villamar M. F. Volz M. S. Bikson M. Datta A. Dasilva A. F. Fregni F. (2013). Technique and considerations in the use of 4x1 ring high-definition transcranial direct current stimulation (HD-tDCS). *J. Vis. Exp.* 77:e50309 10.3791/50309 PMC3735368 23893039

[B73] Weber B. J. Huettel S. A. (2008). The neural substrates of probabilistic and intertemporal decision making. *Brain Res.* 1234 104–115. 10.1016/j.brainres.2008.07.105 18710652 PMC2629583

[B74] Weller J. A. Levin I. P. Bechara A. (2010). Do individual differences in Iowa Gambling Task performance predict adaptive decision making for risky gains and losses? *J. Clin. Exp. Neuropsychol.* 32 141–150. 10.1080/13803390902881926 19484643

[B75] Weygandt M. Mai K. Dommes E. Leupelt V. Hackmack K. Kahnt T. (2013). The role of neural impulse control mechanisms for dietary success in obesity. *Neuroimage* 83 669–678. 10.1016/j.neuroimage.2013.07.028 23867558

[B76] Wittmann M. Paulus M. P. (2008). Decision making, impulsivity and time perception. *Trends Cogn. Sci.* 12 7–12. 10.1016/j.tics.2007.10.004 18042423

[B77] Xu L. Liang Z. Y. Wang K. Li S. Jiang T. (2009). Neural mechanism of intertemporal choice: from discounting future gains to future losses. *Brain Res.* 1261 65–74. 10.1016/j.brainres.2008.12.061 19185567

[B78] Yechiam E. Busemeyer J. R. Stout J. C. Bechara A. (2005). Using cognitive models to map relations between neuropsychological disorders and human decision-making deficits. *Psychol. Sci.* 16 973–978. 10.1111/j.1467-9280.2005.01646.x 16313662

[B79] Yechiam E. Kanz J. E. Bechara A. Stout J. C. Busemeyer J. R. Altmaier E. M. (2008). Neurocognitive deficits related to poor decision making in people behind bars. *Psychon. Bull. Rev.* 15 44–51. 10.3758/PBR.15.1.44 18605478 PMC3985492

[B80] Zermatten A. Van der Linden M. d’Acremont M. Jermann F. Bechara A. (2005). Impulsivity and decision making. *J. Nerv. Ment. Dis.* 193 647–650. 10.1097/01.nmd.0000180777.41295.65 16208159

