# Supplementary material for: Anodal Stimulation of the Left DLPFC Increases IGT Scores and Decreases Delay Discounting Rate in Healthy Males
Source: Front Psychol. 2016 Sep 20;7:1421. doi: 10.3389/fpsyg.2016.01421 (PMC5028393; doi:10.3389/fpsyg.2016.01421)
Supplement: Supplementary file 1 [file Data_Sheet_1.PDF]

Supplementary Text

Detailed information about the calculation of parameters for the rEV model of IGT and a comparison with an alternative model

Following previous research (He et al., 2012; Yechiam et al., 2005; Yechiam et al., 2008a; Yechiam et al., 2008b), we used the revised expectancy valence (rEV) model to decompose IGT performance into the following three parameters that reflect underlying cognitive and emotional processes.

1) Reward sensitivity (w)

w is a weight parameter that represents relative attention to gains over losses (i.e., reward sensitivity) in the following equation.

$$v(t) = w * win(t) - (1\text{-}w) * loss(t)$$

where for a given trial (*t*), *v(t)* represents the valence of the payoffs experienced after choosing a deck, *win(t)* the amount of money received, and *loss(t)* the amount lost. w ranges from 0 to 1, with higher values denoting increased attention to gains over losses.

2) Recency (ϕ)

Recency represents relative attention to the most recent outcome over earlier outcomes, as formulated in the following equation.

$$E_j(t) = E_j(t-1) + \emptyset \times \left[v(t) - E_j(t-1)\right]$$

where for a given trial (*t*), expectancy (*E*) for a given deck (*j*) is updated as a function of previous expectancy with an adjustment. Recency (ϕ) ranges from 0 to 1, with higher values indicating more rapid discount of past outcomes.

3) Choice consistency (*c*).

According to the rEV model, the probability of choosing a card from a given deck (j) in trial t+1 (Pr[Gj(t+1)]) is calculated as the strength of that deck relative to the sum of the strengths of all decks:

$$\Pr\left[G_j(t+1)\right] = \frac{e^{\theta(t)\times E_j(t)}}{\sum_k e^{\theta(t)\times E_k(t)}}$$

where θ(t) represents the consistency between the expectancies and the actual choices, and is further defined as a power function for the sensitivity change over trials.

$$\theta(t) = (t/10)^c$$

where *c* is the Choice consistency parameter, ranging from -5 to 5, with higher values representing converging choices toward the decks with the maximum reward expectancy and lower values representing erratic choices (i.e., inconsistent with the expectancies).

In addition, following previous studies (Yechiam & Busemeyer, 2008; Yechiam et al., 2008b), we compared the rEV model with a simple statistical model that predicts the next choice solely on the basis of the average choice proportions. Difference of the Bayesian Information Criterion (dBIC) was used to compare the relative fit of models.

dBIC = 2*log likelihood difference -k*ln(N)

where k represents the difference between models in the number of parameters and N the number of observations (100). The log likelihood difference was calculated with Formula 9 in Yechiam and Busemeyer's (2008) paper. The rEV model analysis script can be freely downloaded from the following link:

http://ie.technion.ac.il/~yeldad/Expectancy_valence_constanc_example.zip

A positive dBIC value (i.e., > 0) would indicate that the rEV model performed better than the alternative model (**Table S1**). The dBIC values of the two groups/conditions were also compared to see if the model fit the two conditions differently (**Table S1**).

Supplementary Table

Table S1 Mean dBIC of the rEV Model as Compared to the Baseline Model

Experiment	tDCS	Sham	Comparison Between Conditions
1	18.39 ± 4.67***	18.58 ± 4.83***	$t(39) = 0.13$, $p = 0.55$
2	16.68 ± 4.77***	16.53 ± 4.29***	$t(47) = 0.12$, $p = 0.55$
3	18.83 ± 4.32***	18.68 ± 4.52***	$t(19) = 0.16$, $p = 0.56$

***$p < .001$ based on one-sample t test against no difference between the rEV model and the baseline model. Significant positive t values (> 0) suggested that the rEV model performed better than the baseline model. Differences in dBIC between the two conditions/groups (tDCS vs. Sham) were tested with two-sample t test and the results are shown in the third column.

References

1. He, Q., Xue, G., Chen, C., Lu, Z.L., Chen, C., Lei, X., Liu, Y., Li, J., Zhu, B., Moyzis, R.K., Dong, Q., Bechara, A., 2012. COMT Val158Met polymorphism interacts with stressful life events and parental warmth to influence decision making. Sci Rep 2, 677.
2. Yechiam, E., & Busemeyer, J. R. 2008. Evaluating generalizability and parameter consistency in learning models. *Games & Economic Behavior*,*63*(1), 370-394.
3. Yechiam, E., Busemeyer, J.R., Stout, J.C., Bechara, A., 2005. Using cognitive models to map relations between neuropsychological disorders and human decision-making deficits. Psychol Sci 16, 973-978.
4. Yechiam, E., Hayden, E.P., Bodkins, M., O'Donnell, B.F., Hetrick, W.P., 2008a. Decision making in bipolar disorder: A cognitive modeling approach. Psychiatry Research 161, 142-152.
5. Yechiam, E., Kanz, J.E., Bechara, A., Stout, J.C., Busemeyer, J.R., Altmaier, E.M., Paulsen, J.S., 2008b. Neurocognitive deficits related to poor decision making in people behind bars. Psychon Bull Rev 15, 44-51.
